# Evaluation of the clinical value of shear wave elastography for early detection and diagnosis of diabetic peripheral neuropathy: a controlled preliminary prospective clinical study

**DOI:** 10.1186/s12891-022-06085-z

**Published:** 2022-12-22

**Authors:** Can Wang, Huiqin Wang, Yi Zhou, Shiqi Zhang, Meng Huang

**Affiliations:** 1 Department of Ultrasound Medicine, The First Affiliated Hospital of Anhui Medical University, Hefei, 230022 China; 2 Department of Ultrasound Medicine, Dongcheng branch of The First Affiliated Hospital of Anhui Medical University(Feidong Peoples Hospital), Hefei, 231699 China; 3 Department of Endocrinology, The First Affiliated Hospital of Anhui Medical University, Hefei, 230022 China

**Keywords:** Shear wave elastography, Diabetic peripheral neuropathy, High- frequency ultrasound, Tibial nerve

## Abstract

**Purpose:**

This study aims to analyze the clinical application value of shear wave elastography (SWE) technique for early diagnosis of diabetic peripheral neuropathy (DPN).

**Methods:**

Diabetic patients hospitalized in the Department of Endocrinology of the First Affiliated Hospital of Anhui Medical University from August 2021 to February 2022 were enrolled as DPN group (n=38) and non-DPN group (n=35) based on the neurophysiological examination results. 30 healthy subjects were recruited as the control group during the same period. Ultrasound examination of the tibial nerve and related laboratory tests were examined and collected for the total 103 study subjects. Statistical analysis of the collected data, and the receiver operating characteristic(ROC) curve for determination of the optimal cut-off values of mean stiffness of tibial nerve to detect DPN, with determination of area under curve (AUC), specificity, sensitivity, and Youden index.*P* value < 0.05 is considered statistically significant.

**Results:**

Gender, age and BMI differences among three groups were insignificant (*P*>0.05). The difference of serological indicators between DPN and non-DPN groups was also not found (*P*>0.05), whereas longer duration of diabetes was observed in DPN group as compared to non-DPN group. As to the ultra-sound relevant parameters, the cross-sectional area and elastic modulus of the tibial nerve in both lower extremities among these three groups were not significantly different (Oneway ANOVA analysis) although the differences were indeed observed if we compared DPN group exclusively with non-DPN group, or compared non-DPN group with healthy group, or compared DPN group with healthy group (t test). Additionally, the mean elasticity (Emean) cut-off value for the diagnosis of DPN was preferably taken as 67.55 kPa.

**Conclusion:**

SWE has unique advantages in early detection and diagnosis of DPN, which deserve further research.

## Introduction

Diabetes, clinically manifested as hyperglycemia, is a chronic syndrome which is either caused by insulin deficiency (type 1) or insulin resistance (type 2). Diabetes brings a heavy burden to public health and sociology-economic development Approximately 4.6 million patients die each year in China due to diabetic complications [1]. Hyperglycemia can cause chronic damage to human organs, glands, nerves, muscles, and etc. Mona Asghari [2] and Barbara Malicka [3] have reported that high blood sugar impaired the salivary glands, teeth and gums which might cause halitosis, caries, gum lesions and a series of buccal problems. Moreover, this buccal problem can be transmitted vertically from pregnant women to newborns by influencing the establishment of the buccal microbiome of newborns [4]. Mauy Frujuello Mana’s [5] has found that the prevalence of non-alcoholic fatty liver disease in type 2 diabetic patients was as high as 88.6%, of which more than 1/4 of patients had significant liver fibrosis. Hyperglycemia induces the accumulation of sorbitol, causing increased moisture in nerve cells, diffuse swelling of nerves, obscuration of the perineural structures of nerve fascicles, reduced elasticity, increased hardness, leading to a series of peripheral nerve diseases [6] .

Diabetic peripheral neuropathy (DPN), as one of the late complications of diabetes mellitus [7], has an deleterious effect and irreversible onset. When its clinical symptoms manifested, the disease has already developed to an advanced stage [8] and the patient’s quality of life has ever significantly reduced [9]. Therefore, it is deemed that the early detection and diagnosis of DPN can effectively retard the progression of the disease. In this case, nerve conduction study is the preferred method for detecting DPN [10]. The general neurophysiological examination currently used in clinics mainly monitors the action potential of peripheral nerve trunks. Given the fact that the early DPN only affects small nerve fibers, this method couldn’t be a favorable surrogate for early DPN. Besides, this neurophysiological examination is also costly and poorly reproducible [11]. In this end, new technologies for early DPN screening is strong warranted. Another neurophysiological method, high-frequency ultrasound, could observe nerve morphological changes but the morphological specificity and sensitivity are low [12]. Moreover, morphological manifestations could be only easily observed at overt DPN stage, limiting the value of high-frequency ultrasound in the diagnosis of DPN. Shear wave elastography (SWE) is a new ultrasound detection technique and can effectively analyze the elastic changes of the target tissue while observing the tissue morphology [13]. This study aims to explore the applications of SWE for early diagnosis of DPN.

## Methods

### Patients

A total of 73 patients diagnosed with diabetes who were hospitalized in the Department of Endocrinology, the First Affiliated Hospital of Anhui Medical University from August 2021 to February 2022 were enrolled. The inclusion criteria were as follows: ① patients were diagnosed as type 2 diabetes according to the diagnostic criteria proposed in 1998 by the World Health Organization WHO [14]. ② patients read and signed consent forms. All patients completed the relevant laboratory tests, ultrasound examinations and neurophysiological examinations within 1 week after being admitted to the hospital. According to the neurophysiological examination results, the diabetic patients were assigned to two groups. Patients who had positive neurophysiological results were divided in the DPN group; the remaining patients were assigned to the non-DPN group.

A total of 30 healthy non-diabetic individuals were recruited as the control group. The exclusion criteria were as follows: ① type 1 diabetes mellitus; ② hereditary neurological disorders, alcohol-induced neuropathy and neuropathy caused by inflammation, poisoning and drugs; ③ trauma or lumbar spine disease combined with cerebrovascular disease; ④ patients who had serious condition so that unable to cooperate; ⑤ patients and family members refused to cooperate.

The study was approved by the local ethics committee of the hospital.

### Ultrasound of the tibial nerve in both lower limbs

**Fig. 1:**
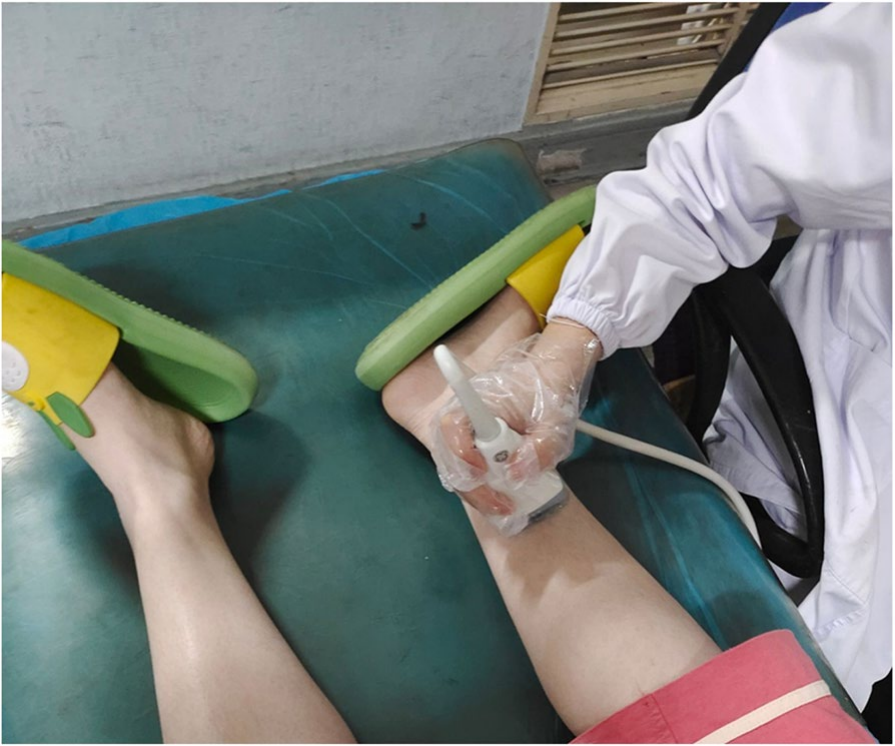
Inspection Demonstration. The patient were asked to fully relax the limbs in supine position and take the natural slight valgus rotation position of both lower limbs so that they could fully expose the medial ankle

All patients were examined within 1 week after being admitted to the hospital by the same ultrasonographer. The ultrasonographer had 7 years experience of using the Supersonic Imagine Aixplorer(0~200kpa), and was blinded to the clinical scoring and nerve conduction test results of the study participants. As to the ultra-sound settings, complete color filling was required in the sampling box and the image after stabilization was needed. A quiet and clean examination environment was then requested. The patient afterwards were asked to fully relax the limbs in supine position and take the natural slight valgus rotation position of both lower limbs so that they could fully expose the medial ankle. The doctor would subsequently acquire the image at 4 cm above the medial ankle which was the cross-section of the tibia nerve at this place and observe whether the tibial nerve indumentum, the echo of the nerve bundle and the internal sieve mesh structure are clear. The image was frozen after the boundary of the nerve was satisfactorily displayed. During measurement, the setting range of elastic modulus value was on, Q box function was enabled, 2 mm in diameter was selected and the elastic modulus of the nerve was measured. All steps were repeated three times and the average value was calculated, as presented in (Fig 1).

### Demographic and clinical data collection

We firstly collected the demographic and clinical information of the three groups including age, body mass index (BMI), gender, and the duration of diabetes. We then measured and collected the serological data including fasting blood glucose (FBG), glycosylated hemoglobin (HbA1C), total cholesterol (TC), triglycerides (TG), and low-density lipoprotein (LDL).

Ultrasonograghy data, including the bilateral tibial nerve cross-sectional area (CSA), right and left diameters, anterior and posterior diameters, tibial nerve elastic modulus under ultrasonography in the three study group were then acquired.

Finally, the sensitivity, specificity, area under the curve (AUC), and Youden index corresponding to Emax, Emean, and Mmin under the ROC curve were calculated to get the best cut-off value for the elastic modulus of the tibial nerve.

### Statistical analysis

SPSS 23.0 software was selected for statistical analysis of all data. Categorical data were described by number of cases or percentage (%). χ^2^ test was used for statistical analysis for comparison of categorical variables. Normally distributed quantitative data were described by mean ± standard deviation. T-test or one-way ANOVA was used for statistical analysis for these data.

The least significant difference(LSD)was selected for comparison after ultra-sound performance. ROC curve for determination of the optimal cut-off values of mean stiffness of tibial nerve to detect DPN, with AUC, specificity, sensitivity, and Youden index. AUC was compared by Z-test. *P* value < 0.05 is considered statistically significant.

## Results

### The comparison of demographic and clinical information

There were 16 males and 22 females in the DPN group with a mean age of (58.61 ±13.87) years. 15 males and 20 females were in the non-DPN group with a mean age of (57.74 ± 14.64) years. Besides, 12 males and 18 females were in the control group with a mean age of (58.40 ±14.32) years. Gender, age and BMI differences among three groups were insignificant (*P*>0.05). The duration of diabetes was significantly longer in the DPN group as compared to the non-DPN group (*P*<0.05). No statistical significance was noted in the differences of FBG, HbA1C, TC, TG and LDL between the DPN and non-DPN groups (*P*>0.05), as shown in (Table 1).

**Table 1:** General information about the study population

	CG (*n* = 30)	DPN (n = 38)	non-DPN (*n* = 35)	*Χ*^*2*^/*F*	*P*
Sex(female/male)	12/18	16/22	15/20	0.057	0.972
Age(yr)	58.40 ± 14.32	58.61 ± 13.87	57.74 ± 14.64	0.036	0.965
BMI((kg/m^2^))	24.38 ± 3.45	24.78 ± 3.77	24.66 ± 3.03	0.117	0.890
Duration(years)	–	11.41 ± 8.240	6.31 ± 5.09	6.057	0.002
FBG(mmol/L)	–	9.25 ± 3.26	8.02 ± 3.48	0.019	0.125
HbA1C(%)	–	9.37 ± 2.22	8.59 ± 1.97	0.557	0.115
TC(mmol/L)	–	4.65 ± 1.01	4.30 ± 1.01	0.004	0.144
TG(mmol/L)	–	1.82 ± 1.21	1.69 ± 1.17	0.007	0.639
LDL(mmol/L)	–	2.90 ± 0.91	2.63 ± 0.84	0.033	0.195

DPN:Diabetic peripheral neuropath; CG: Control group; BMI: Body mass index; FBG:Fasting blood glucose; HbA1C: Gycated hemoglobin glycosylated hemoglobin; TC: Total cholesterol; TG:Triglyceride; LDL: Low Density Lipoprotein

Table 2

**Table 2:** Cross-sectional area of the tibial nerve in both lower limbs

	CSA(cm^2^)			
	LT	RT	*F*	*P*
CG	0.17 ± 0.03	0.16 ± 0.03	0.128	0.411
DPN	0.23 ± 0.03^▲^	0.22 ± 0.03^▲^	0.001	0.400
Non-DPN	0.21 ± 0.03^◆★^	0.21 ± 0.02^◆★^	0.011	0.449
*F*	34.571	36.554	–	–
*P*	<0.001	<0.001	–	–

DPN:Diabetic peripheral neuropath; CG: Control group; CSA: Cross-sectional area; LT:Left tibial nerve; RT: Right tibial nerve

Note: ▲: Statistically significant difference between the control group and the DPN group(*P* < 0.05); ◆: Statistically significant difference between the DPN group and the non-DPN group(*P* < 0.05); ★: Statistically significant difference between the control group and the non-DPN group(*P* < 0.05)

### The comparison of the cross-sectional area

The differences of bilateral lower limb tibial nerve CSA were not statistically significant in any of the three study groups for the within-group comparison (P > 0.05). However, the differences reached statistically significance when comparing the cross-sectional area of the bilateral lower limb tibial nerve exclusively between DPN group and non-DPN group, or between non-DPN group and healthy group, or between DPN group and healthy group(*P* < 0.05), as shown in.

### The comparison of elastic modulus

**Fig. 2:**
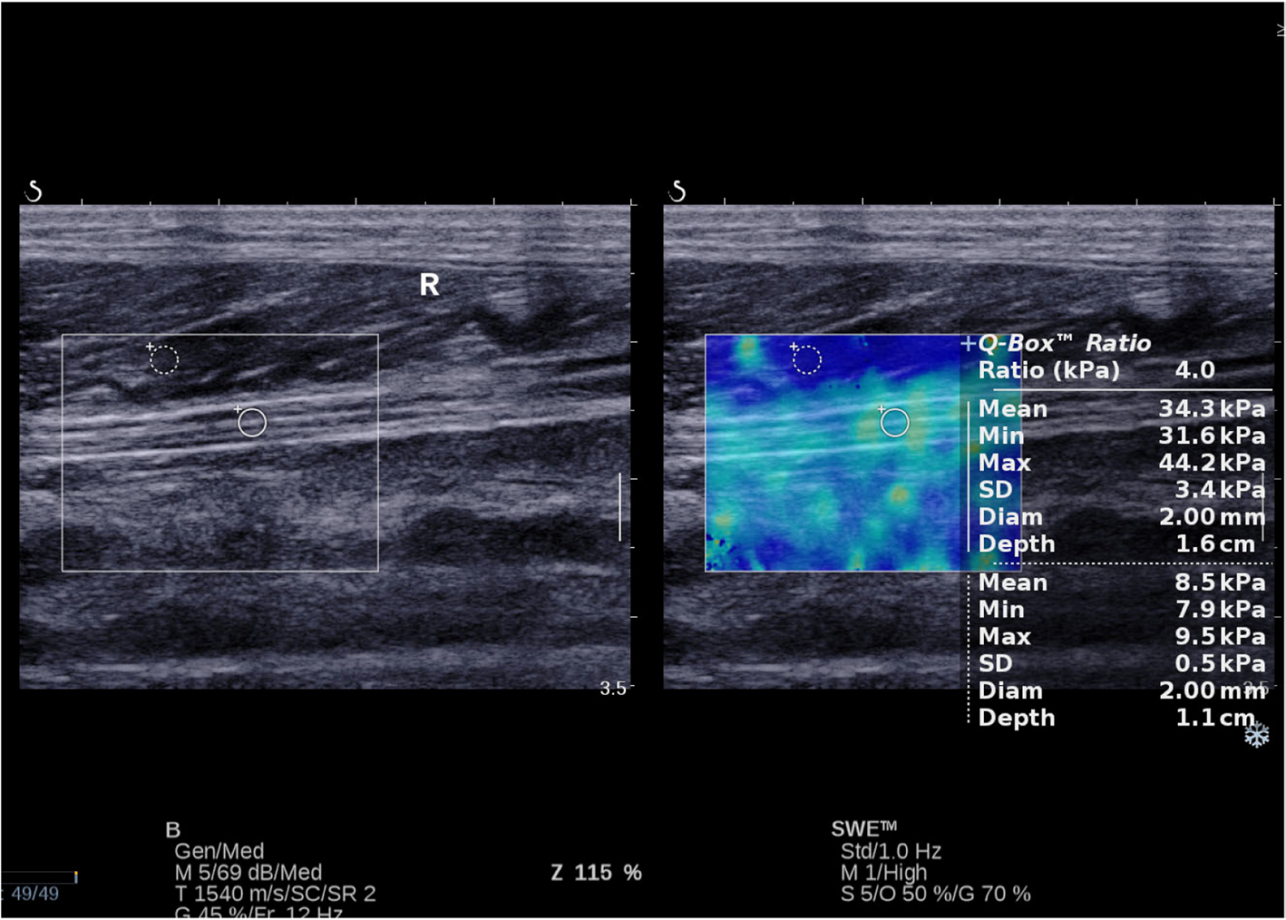
Here was an example of a healthy 63 years old female for the SWE measurement. She had an Emean at 34.3 kPa; an Emin at 31.6 kPa and an Emax at 44.2 kPa in the right lower limb tibial nerve

**Fig. 3:**
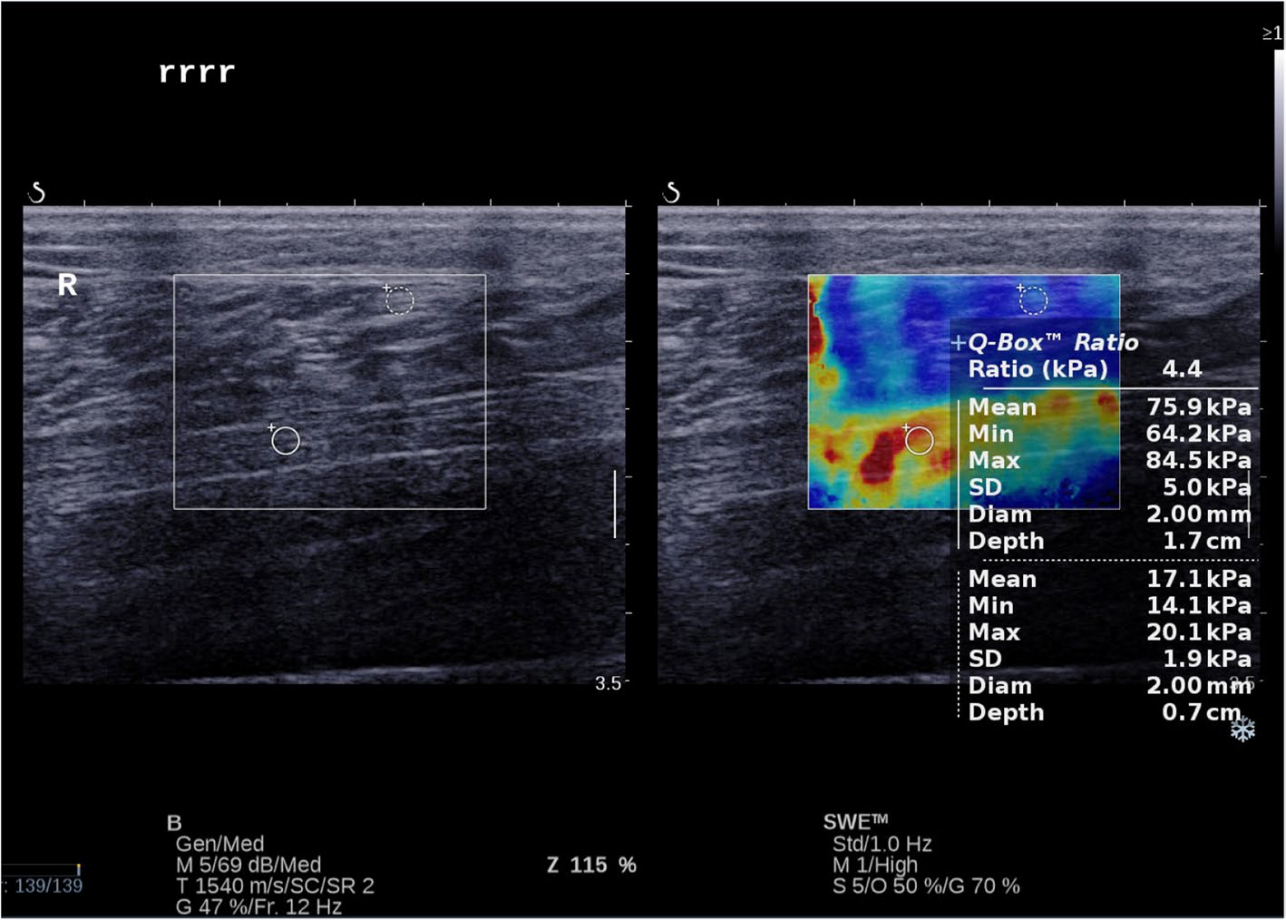
This was an example of a 66 years old female DPN patient for the SWE measurement. She had an Emean at 75.9 kPa, an Emin at 64.2 kPa and an Emax at 84.5 kPa in the right lower limb tibial nerve

**Fig. 4:**
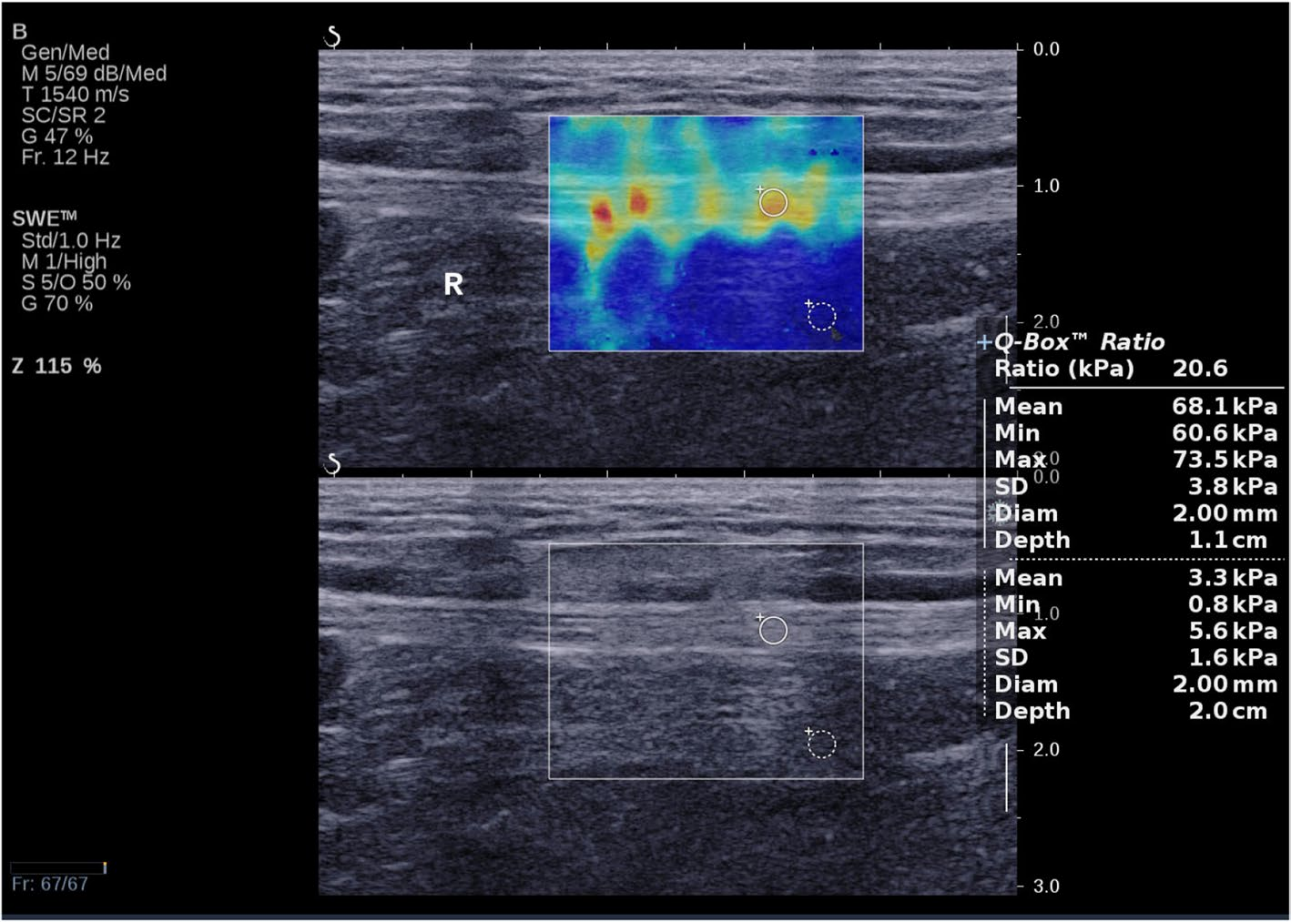
This was an example of a 68 years old male non-DPN patient for the SWE measurement. He had an Emean at 68.1 kPa, an Emin at 60.6 kPa and an Emax at 73.5 kPa in the right lower limb tibial nerve

The within-group comparisons demonstrated that elastic modulus were not statistically significant within three groups (*P* > 0.05). If t-test analysis between each two groups were taken into account, the DPN group however indeed had higher elastic modulus than the non-DPN group and the control group (*P* < 0.05); the non-DPN group had further higher elastic modulus than the control group (P < 0.05), as seen in (Table 3, Figs 2 and 3, 4).

**Table 3:** Elastic modulus of the tibial nerve in both lower limbs

	Emean(kPa)			Emin(kPa)			Emax(kPa)		
	LT	RT	P	LT	RT	P	LT	RT	P
CG	45.94 ± 6.97	44.43 ± 6.57	0.390	36.88 ± 7.19	35.74 ± 11.81	0.653	59.50 ± 6.87	56.28 ± 8.84	0.121
DPN	73.26 ± 7.96	73.80 ± 9.16	0.786	56.37 ± 9.12	55.44 ± 9.35	0.663	84.66 ± 9.71	83.97 ± 9.68	0.756
non-DPN	64.60 ± 8.87	66.14 ± 8.29	0.456	49.23 ± 9.60	49.36 ± 10.42	0.956	76.95 ± 8.12	76.36 ± 10.83	0.795
P_(CG vs DPN)_	*P* < 0.001	P < 0.001	–	*P* < 0.001	P < 0.001	–	P < 0.001	P < 0.001	–
P_(CG vs non-DPN)_	P < 0.001	P < 0.001	–	P < 0.001	P < 0.001	–	P < 0.001	P < 0.001	–
P_(DPN vs non-DPN)_	P < 0.001	P < 0.001	–	0.001	0.015	–	P < 0.001	0.001	–

DPN:Diabetic peripheral neuropath; CG: Control group; Emax: Maximum elasticity; EMean: Mean elasticity; EMin: Minimum elasticity; LT:Left tibial nerve; RT: Right tibial nerve

### Receiver operating characteristic curve

**Fig. 5:**
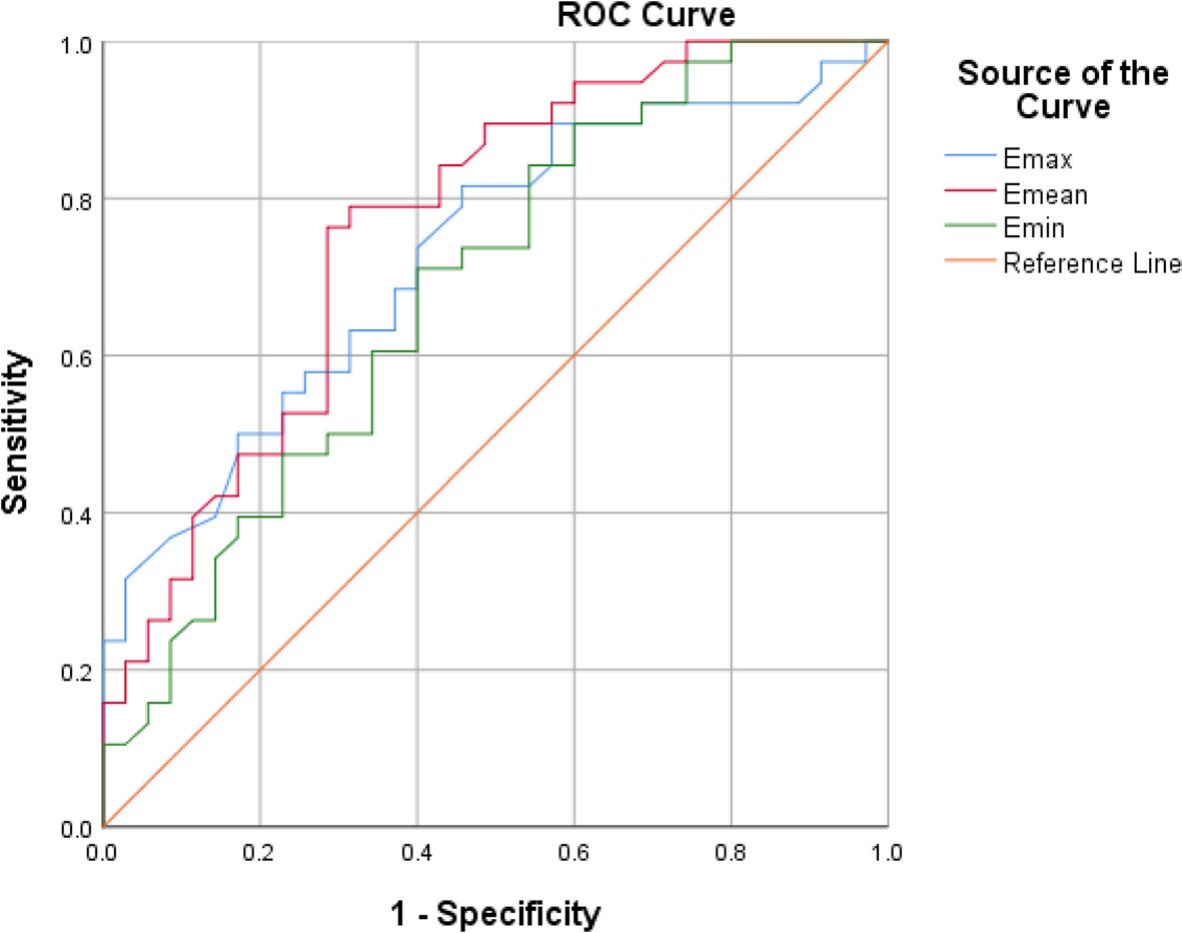
ROC curve for the diagnosis of DPN based on SWE

ROC curve was plotted according to the data obtained. The sensitivity, specificity, AUC and Youden index were thus calculated.For Emean, the AUC of 0.763 at an optimal cut-off value was 67.55 kPa; the sensitivity was 76.3%; the specificity was 71.4% and the Youden index was 0.477. For Emax, the AUC of 0.728 at an optimal cut-off value was 77.75 kPa; the sensitivity was 81.6%; the specificity was 54.3% and the Youden index was 0.359. For Emin, the AUC of 0.685 at an optimal cut-off value was 52.80 kPa; the sensitivity was 71.1%; the specificity was 60.0% and the Youden index was 0.311. Thereby, the Emean optimal cut-off value of 67.55 kPa was found to be of the optimum diagnostic value of DPN (Fig 5).

## Discussion

SWE, a new ultrasound technique, is used to assess the stiffness of target tissues by emitting transient pulses to generate shear waves. Subsequently, the transverse shear wave velocity is obtained by intelligent computer analysis. The process is colour coded into easily observable colour images based on that velocity [15]. In 2010, scholars [16] first proposed the application of SWE for the prediction of benign and malignant thyroid tumors. Hereafter, SWE was successively applied to the assessment of hardness and elasticity of various tissues such as thyroid, breast, liver, muscle, and bone. Later, in 2013 Fatih Kantarci [17] suggested that SWE can be utilized in the diagnosis of temporomandibular disorders(TMD)since it was highly reproducible in the assessment of median nerve hardness. This conclusion was subsequently again verified by another study in 2019 which demonstrated a decrease in anterior malleolar disc stiffness of less than 8.667 kPa could accurately identify patients with TMD [18]. During the same period, Gürün E [19] proposed that the use of SWE examination to find brachial plexus nerve roots in patients with Multiple sclerosis has an increase in elasticity values and a decrease in diameter values, and his findings are consistent with the demyelinating process of the peripheral nervous system due to Multiple sclerosis.In 2016, Atilla Suleyman Dikici et al. [20] first proposed the application of SWE to the detection of DPN and they found that increased stiffness of the tibial nerve in the lower limbs could be a surrogate for the diagnosis of DPN with high sensitivity and specificity. Recently, SWE was furthermore found to be potential as a non-invasive test instead of liver biopsy in children with non-alcoholic fatty liver disease [21]. The stiffness of the occlusal muscles of patients during treatment of masticatory muscle disorders could also be assessed by SWE as interpreted by Anna Olchowy in 2022 [22]. SWE is widely used in nerve and muscle research.

Our study showed that the duration of disease was longer in the DPN group than in the non-DPN group, suggesting a correlation between the duration of disease and the development of DPN which was consistent with previous studies [23]. In terms of ultrasound parameters, although the differences of bilateral lower limb tibial nerve CSA were not statistically significant among the three study groups, the differences were found if we compared the parameter between each two groups. The latter comparison showed that DPN group had higher bilateral lower limb tibial nerve CSA than both non-DPN group and healthy group, supporting the diagnosis of DPN by SWE [24]. The study of high-frequency ultrasound was in line with this conclusion. It might be explained by the enlarged diameter and CSA of nerves,with obscuration of the perineural structures of nerve fascicles, and decreased echoes of nerve fibers in diabetic patients. The mechanisms of these morphological change could be related to hyperglycemia-induced nerve edema, demyelination and axonal degeneration [25].

Not only nerve CSA but also the elastic modulus was evaluated in our study since the nerve CSA is influenced by multiple factors such as sex, weight, etc. In our study, the DPN group displayed higher elastic modulus than non-DPN group. The latter group furthermore displayed higher elastic modulus than the control group, suggesting that the involvement of reduced nerve elasticity and increased stiffness in DPN diagnosis.

Based on the ROC curve, our data deciphered Emean was higher (approximately 71.4%) than EMin (60%) and EMax (54.3%) in terms of specificity while Emax and Emean (81.6 and 76.3% respectively) were more sensitive in terms of sensitivity. With an Emean optimum cut-off value of 67.55 kPa the AUC was 0.765, which had the highest diagnostic value. Which is broadly consistent with Ibrahim Heba R ‘s findings [26]. Based on the optimal cut-off values obtained from the ROC curve, we retrospectively concluded as follows. Firstly, the control group had normal nerve elasticity of tibial nerves in both lower limbs. Secondly, the average elastic modulus of the DPN group was much higher than the optimal cut-off value, indicating that the bilateral lower limb tibial nerve had been impaired. Lastly, the average elastic modulus of the non-DPN group was close to the optimal cut-off value. As an example, the Emean value of the patient’s lower extremity tibial nerve SWE in Fig 4 was 68.1 kPa. Based on the optimal threshold value (67.55 kPa), the patient was already affected by neuropathy. In the light of this, it could be speculated that although no neuropathy was detected in the non-DPN group with neurophysiological tests there were some patients already at the early stage of DPN. Taken together, SWE could be much more valuable for the early diagnosis of DPN as it could detect the onset of neuropathy before the abnormal neurophysiological results appeared [27].

The major limitation of our study is the small group size. In addition, the data concerning DPN symptoms were not collected in our present study although most DPN patients indeed showed DPN symptoms such as pain and/or numbness in the upper or lower extremities or their physical examinations suggested a decline in temperature perception, vibration and/or position sensation. Yet it should be emphasized that this study used a cross-sectional design and therefore confirmation of this assumption should ideally be prospectively followed-up in a larger longitudinal study with the inclusion of DPN symptoms.

## Conclusions

SWE is an emerging ultrasound technology that meets the requirements of early DPN screening with real-time, simplicity, non-invasiveness and repeatability. At the same time its functional assessment of nerves compensates for the shortcomings of traditional high-frequency ultrasound in monotonically observing nerve alignment and morphology, which achieves a comprehensive assessment of nerves and has unique advantages in the early detection and diagnosis of DPN.

## Data Availability

The dataset generated and analyzed during the current study are not publicly due to privacy restrictions but available from the corresponding author upon reasonable request.

## Abbreviations

DPN: Diabetic peripheral neuropath
SWE: Shear wave elastography
BMI: Body mass index
FBG: Fasting blood glucose
HbA1C: Gycated hemoglobin |glycosylated hemoglobin
TC: Total cholesterol
TG: Triglyceride
LDL: Low Density Lipoprotein
CSA: Cross-sectional area
ROC: Receiver operating characteristic curve
AUC: Area under the curve of ROC
LSD: Least significant difference
Emax: Maximum elasticity
EMean: Mean elasticity
EMin: Minimum elasticity
CG: Control group
LT: Left tibial nerve
RT: Right tibial nerve
